# 

**Published:** 1885-07

**Authors:** 


### OUR ADVERTISERS.

ATTENTION is directed to the advertisements of the Medical Colleges in this issue.

PEACOCK'S BROMIDES, AND FUCUS MARINA, made by the Peacock Chemical Co., of St. Louis, are worthy of a trial. See advertisement and send for sample.

THE CINCINNATI SANITARIUM, at College Hill, Ohio, a private hospital for the insane, is an institution worthy the confidence of the profession. See advertisement, page 20, and write for information.

Dr. N. Sypert, Laurel Hill, Pa., says: "I have been using lactopeptine for two years in my practice, and so far it has proved successful in cholera infantum, dysentery, and all diseases of the bowels."

DRS. TALIAFERRO & NOBLE, ATLANTA.—We take pleasure in calling attention to the advertisement of these gentlemen, to be found on page 3. Their infirmary for the treatment of diseases of women is a first-class institution in every respect and deserves the success it has attained.

MAGNUS & HIGHTOWER, ATLANTA.—No firm in the city has made more steady progress than this one. They are young men who began business here a little more than a year ago as retail dealers and prescriptionists. They have now one of the largest wholesale drug stores to be found in the South, and have recently purchased the handsome retail drug store on corner of Peachtree and Decatur streets. They are better prepared than ever before to serve their numerous customers, both in and out of the city. See their attractive advertisement on page 6, and write them for anything you may need in their line.

